# Off-Label Promotion, On-Target Sales

**DOI:** 10.1371/journal.pmed.0050210

**Published:** 2008-10-28

**Authors:** Adriane Fugh-Berman, Douglas Melnick

## Abstract

Adriane Fugh-Berman and Douglas Melnick describe techniques by which pharmaceutical companies covertly promote off-label drug use even where such promotion is illegal.

For prescription drugs, demonstrated benefits define the parameters of acceptable risks. For example, liver toxicity may be acceptable in a drug approved for cancer, but unacceptable in a drug approved for acne. Government regulatory bodies review laboratory, animal, and human data to confirm that a drug has the claimed efficacy and safety prior to approving its release in the market for specific approved (“labeled”) uses.

Once a drug is approved for at least one indication, it may be prescribed off-label for a different condition, a different population, or in a different dose than what the drug is approved for. However, off-label uses have not been subject to the testing and review that is a precondition for marketing approval. The scientific review of evidence of effectiveness and safety that regulators weigh prior to approval for a labeled indication protects the patient. With off-label use, this protection often does not exist.

Off-label prescription of a drug is generally legal, but promotion of off-label uses by a drug manufacturer is usually illegal. This paper addresses public health issues associated with off-label use, and describes techniques by which pharmaceutical companies covertly promote off-label use even where such promotion is illegal.

## Risks and Benefits for Patients

Off-label use is sometimes unavoidable; three-quarters of marketed prescription drugs have no labeling indications for children, a population only recently included in clinical trials [1]. Pregnant women are also routinely excluded from studies, so most drug treatment during pregnancy is off-label.

Some off-label use is demonstrably beneficial. For example, in the United States, misoprostol, a prostaglandin, is approved only to prevent ulcers. However, misoprostol is widely used off-label for ripening the cervix, inducing abortion, and other indications, and more than 200 studies involving more than 16,000 pregnant women support the use of misoprostol in obstetrics [2].

Pregnant women and children, however, do not account for most off-label use. In 2001, 150 million off-label prescriptions were written—21% of all prescriptions written for 160 common medications in the US. About three-quarters (73%) of off-label prescriptions were written for conditions for which there was little or no scientific support for efficacy [3]. Up to 75% of drug use in cancer care, and about 90% of drug use in rare diseases, is off-label [1].

Off-label use of drugs has been associated with serious adverse effects. For example, Duract (bromfenac), an analgesic, was approved only for treating acute pain, and only for short-term use (less than ten days). However, some physicians prescribed Duract off-label for longer durations. Duract caused liver failure, and was withdrawn from the market less than a year after approval [4]. The appetite suppressant Pondimin (fenfluramine), approved for short-term use, was widely prescribed with phentermine and used long-term. The off-label combination “fen-phen” caused valvular heart disease [4,5]. In children, off-label use of drugs is associated with an increased number and severity of adverse effects [6].

## Risks—and Benefits—for Profits

From a business standpoint, increased off-label use means larger revenues from larger user populations, especially for products with narrow indications. For example, a company that knows that an approved leukemia drug reduces facial wrinkles could fund an efficacy trial in people with wrinkles in order to garner a new indication. However, clinical trials are expensive, and the results could decrease sales by showing that the drug is ineffective, or has significant safety problems. A company-funded long-term trial that tested the efficacy of Vioxx (rofecoxib) for colon polyps turned up cardiovascular risks that eventually resulted in the drug being withdrawn [7].

The disadvantage of an off-label market is limited growth, because a company cannot legally promote sales. Warner-Lambert, a subsidiary of Pfizer, paid US$430 million in criminal fines and civil payments for off-label promotion of Neurontin (gabapentin) [8].

Although off-label use is usually advantageous for companies, occasionally it works against company goals. For example, despite robust evidence for the safety and efficacy of misoprostol in obstetrics, the manufacturer, perhaps not wanting to be associated with an abortion drug, would not seek approval from the US Food and Drug Administration (FDA) for any reproductive health uses [9]. Publicly funded studies were eventually performed [10].

Genentech found itself in the awkward position of battling off-label use of its own product. After Lucentis (ranibizumab) was approved to treat age-related wet macular degeneration, ophthalmologists quickly substituted Genentech's Avastin (bevacizumab), a similar, cheaper drug approved for cancer treatment. While arguing that no trials supported bevacizumab for age-related wet macular degeneration, Genentech refused to conduct comparative trials, presumably because a finding of equivalency would undercut sales of the more expensive drug. The National Eye Institute of the National Institutes of Health plans a trial comparing the two drugs [11].

## How To Promote Off-Label

One of us (DM) worked in the pharmaceutical industry as a physician in medical affairs, supporting marketing, for over five years, and both authors have current contacts within the industry. Any unreferenced material that follows is from our personal experience, contacts, or information available in public fora such as industry discussions and presentations at trade shows.

In development, drugs may be promising for several uses, and companies must choose one or two conditions on which to focus research. Ease of approval is the most important factor in this decision. If extensive off-label use is anticipated, a company may seek approval for a narrow indication in order to speed a drug to market. In other words, a drug may be approved for a decoy indication while an extensive off-label campaign is not disclosed to regulators. For example, a company that plans to promote a drug off-label for cancer prevention could avoid the costs and delay that a long-term trial entails by instead funding a relatively inexpensive trial of ulcer treatment, or—even better for business—rabies. Rabies is rare, and in the US, treatments for rare diseases are “orphan drugs,” eligible for expedited six-month FDA reviews. Orphan drugs enter the market faster.

Once a drug is approved for a decoy indication, labeled and off-label promotion may occur concurrently. Journal advertising and direct mail channels are used to market labeled indications. Off-label campaigns are launched outside of the sales force.

Nationally known, influential academic physicians help “word-of-mouth” or “buzz” marketing. These “thought leaders” or “key opinion leaders” (KOLs) support labeled marketing efforts as well, but they are considered crucial for the promotion of off-label uses. Industry-paid KOLs are never company employees. Rendering purportedly independent opinions, via articles and lectures, KOLs are able to elude laws against off-label promotion.

## Commentaries and Case Studies

In the pharmaceutical industry, there are two ways to market an approved drug for a new use: the “indication” route—performing studies necessary for regulatory approval—or the “publication” strategy, which stimulates off-label prescribing by using research “to disseminate the information as widely as possible through the world's medical literature” [12].

Clinical studies provide key references for the industry-produced reviews and commentaries, signed by KOLs, used for promoting off-label sales. Case studies about off-label uses may be solicited; physicians may be paid for combing patient medical records for cases that help industry goals. A physician—or a medical writer—will write up the case or case series, which may be submitted for publication or presented as a meeting abstract. Industry-sponsored reprints may be included in continuing medical education (CME) activities sponsored by medical education companies (MECs), often distributed by direct mail.

## Marketing via Meetings: Abstracts and Posters

Posters and abstracts presented at medical meetings create buzz, especially if a press release garners media attention. Meeting abstracts and posters are considered cutting-edge, but the information is almost always incomplete and usually lacks peer review. While abstracts must be submitted six months before meetings, posters can be altered, without review, up to the day of the meeting. Poster reproductions are made available to meeting attendees, and are an ideal form of stealth marketing.

Abstracts or posters may be “published” in conference proceedings, medical journals, “throwaway” journals, or industry-sponsored medical journal supplements. These industry-generated, non-peer-reviewed, covert promotional pieces are now citable items that are provided to physicians by a company's medical affairs office to support off-label use, and can be referenced in peer-reviewed articles, ads, and other marketing materials.

Industry-sponsored abstracts, posters, and publications are designed to serve marketing purposes and so must foster positive impressions of targeted drugs. If a poster or abstract generates positive buzz, study publication is unnecessary, and could even hurt sales. For example, a poster might present preliminary results of a study that showed promising effects of a drug in 50 patients. Perhaps, after 200 patients finished the study, the drug proved no better than placebo. A company could then decide against publishing the trial. Doctors exposed to positive preliminary results and protected from negative final results would still regard the drug favorably.

## Medical and Graduate Education

Publications and posters provide the foundation for the medical education programs that are key for promoting off-label uses [12,13]. “Medical education drives this market!” stated a Parke-Davis business plan revealed in a legal case regarding off-label promotion [12]. MECs know that accredited CME programs funded by unrestricted grants must favor marketing messages. The easiest way to accomplish this goal is to use company speakers trained for unaccredited (non-CME) promotional presentations.

Physician-speakers are trained in presenting unaccredited talks, sometimes called “dinner talks” or “lunch-and-learns.” This training, using company slides, often occurs at resorts. Some speakers are genuinely unaware of the marketing messages they are responsible for disseminating. For example, messages that a certain disease is underdiagnosed, undertreated, or more serious than commonly believed can bolster a company's marketing goals even if drugs are never mentioned.

Physicians trained in unaccredited talks may present the same talk at a CME event. Most industry-paid physicians believe that they maintain intellectual independence. Presenting different statements in different settings would create cognitive dissonance. Psychologically, it is easier to believe that what one is saying is scientific and accurate, and thus to say the same thing at accredited and unaccredited programs.

“Unrestricted” grants provided to departments at academic medical centers for grand rounds and lunch conferences depend on a sense of obligation rather than a quid pro quo. When lists of recommended speakers are supplied to organizers, it is unstated, but nonetheless understood, that company-paid speakers will be included in the lecture series.

Although company-provided materials usually do not recommend off-label use, speakers may modify the slides, or simply address off-label uses verbally. Companies are supposed to stop using speakers who consistently promote off-label uses, but that may not happen.

## The Role of Reps

In the US, although pharmaceutical representatives are not supposed to detail doctors on off-label uses, representatives are rated, and compensated, based on sales. As one industry consultant quoted in *Medical Marketing and Media* stated, “Let's say the sales goal [for a drug] is larger than if every patient over 60 is already on it. Divide that down to territories, and everybody has to meet it. The message is, sell off-label.” A pharmaceutical industry attorney quoted in the same article stated, “Before engaging in off-label promotion, companies should ascertain the risk profile, safety, efficacy, and potential commercial benefits of the use—without committing that last bit to print” [14]. In other words, illegal promotion may be cost-effective if potential profits trump potential fines.

Companies distance themselves from “rogue” reps caught promoting drugs off-label, but in practice, monitoring reps may be bad for the bottom line. Some marketing information companies track rep behavior, usually by interviewing physicians, and sell this information to pharmaceutical companies interested in the behavior of their own—or competing—reps. Some companies, however, ask that potential negative information about their own reps be removed before the information is supplied [15].

Skilled pharmaceutical representatives can solicit questions about off-label use from doctors. After “cueing” the doctor to ask an off-label question, the rep can fill out a postcard or call the company to send a packet of off-label information from the medical affairs office. The packet may contain company-approved reprints and a “standardized letter,” created by the drug information department of the company, discussing any research on the off-label use. The US is considering loosening restrictions on the distribution of reprints regarding off-label uses by pharmaceutical representatives. In February 2008, the FDA released a draft guidance for industry that would allow distribution of reprints from peer-reviewed publications with genuine editorial boards.

## Coverage through Compendia

Compendia are compilations of drug information that include both on-label and off-label uses. Medicare, Medicaid, and many other insurers will cover off-label uses of reimbursable drugs included in major compendia, including the American Hospital Formulary Service–Drug Information (American Society of Health-System Pharmacists), the US Pharmacopoeia–Drug Information (Micromedex, a division of the Thomson Publishing Company), and DRUGDEX. Pharmaceutical companies strive to establish good relationships with compendia staff, and may assign an employee as the designated compendia contact. The pharmacists who write compendia listings are very busy, and are usually delighted to receive organized packets of scientific articles, abstracts, and contact information. Using company-provided articles (which always contain marketing messages) saves time; all of the company's assertions for off-label use may be transferred intact to the final product. Companies celebrate new compendia listings because expanded insurance coverage ensures more sales.

Drug information listings, similar to compendia, usually solicit company input and review. Although companies do not pay for listings, they may offer to buy hundreds or thousands of reprints for sales staff. In any case, it makes sense to contribute to entries that show company data in the best light.

## Discussion/Conclusions

While off-label use is sometimes necessary, it should be undertaken with care and caution due to the uncontrolled experiment to which a patient is being subjected. Valuable off-label uses should be discussed by unbiased researchers in bona fide medical journals. Promising therapies should be tested in clinical trials. Truly useful off-label benefits of drugs will not remain a secret.

Pharmaceutical companies cannot be expected to police off-label promotion by sales representatives when such promotion financially benefits both the reps and companies. Washington D. C. recently became the first jurisdiction to require licensure of pharmaceutical sales representatives. The SafeRx Act requires drug reps to be held to a professional code of conduct, prohibits them from engaging in deceptive or misleading marketing, and requires continuing education as a requirement for license renewal [16]. If the city monitors and punishes infractions, this novel approach could mitigate off-label promotion.

States and other jurisdictions have a duty to protect the health of the public. Allowing off-label promotion of drugs for untested, unproven benefits maximizes industry profits at the expense of public health. A risk–benefit ratio cannot be assessed without knowing whether benefits exist. Where no benefits exist, no risk is acceptable.

Pharmaceutical marketing has distorted the discourse on off-label uses and encouraged the unmonitored, potentially dangerous use of drugs by patients for whom risks and benefits are unknown. Companies that engage in off-label promotion should be heavily fined and their future marketing practices subject to increased scrutiny by regulatory agencies. Perhaps financial incentives could be provided to reward physicians and others who report off-label promotion. Such programs would target only industry representatives, not clinicians, and would be easily covered by industry fines. Restrictions on off-label promotion of drugs should be strengthened, not gutted.

## Abbreviations

CME: continuing medical education
FDA: Food and Drug Administration
KOL: key opinion leader
MEC: medical education company
